# Supplementary material for: A UMLS-based spell checker for natural language processing in vaccine safety
Source: BMC Med Inform Decis Mak. 2007 Feb 12;7:3. doi: 10.1186/1472-6947-7-3 (PMC1805499; doi:10.1186/1472-6947-7-3)
Supplement: Additional file 13 — RAP application directory. Sets up directory for RAP (RDF application for PHP) source code [file 1472-6947-7-3-S13.gz › rap/doc/phpdoc/__filesource/fsource_default__vocabularyRSS_C.php.html]

xml version="1.0" encoding="iso-8859-1"?


File Source for RSS\_C.php


# Source for file RSS\_C.php

Documentation is available at RSS\_C.php

```
1. <?PHP
2. // ----------------------------------------------------------------------------------
3. // RSS Vocabulary (Resource)
4. // ----------------------------------------------------------------------------------
5. // Version                   : 0.9
6. // Authors                   : Tobias Gau� (tobias.gauss@web.de)
7. //
8. // Description               : Wrapper, defining resources for all terms of RSS.
9. //                               For details about RSS see: http://purl.org/rss/1.0/.
10. //                                Using the wrapper allows you to define all aspects of
11. //                             the vocabulary in one spot, simplifing implementation and
12. //                             maintainence.
13. //
14. // ----------------------------------------------------------------------------------
15. // History:
16. // 11-08-2003                 : Initial version
17. // ----------------------------------------------------------------------------------


25. class RSS{

27. function CHANNEL()
28. {
29. return  new Resource(RSS_NS . 'channel');

31. }

33. function IMAGE()
34. {
35. return  new Resource(RSS_NS . 'image');

37. }

39. function ITEM()
40. {
41. return  new Resource(RSS_NS . 'item');

43. }

45. function TEXTINPUT()
46. {
47. return  new Resource(RSS_NS . 'textinput');

49. }

51. function ITEMS()
52. {
53. return  new Resource(RSS_NS . 'items');

55. }

57. function TITLE()
58. {
59. return  new Resource(RSS_NS . 'title');

61. }

63. function LINK()
64. {
65. return  new Resource(RSS_NS . 'link');

67. }

69. function URL()
70. {
71. return  new Resource(RSS_NS . 'url');

73. }

75. function DESCRIPTION()
76. {
77. return  new Resource(RSS_NS . 'description');

79. }

81. function NAME()
82. {
83. return  new Resource(RSS_NS . 'name');
84. }
85. }


89. ?>
```

Documentation generated on Fri, 17 Dec 2004 16:18:07 +0100 by phpDocumentor 1.3.0RC3
